# Efficacy of a New Medical Information system, Ubiquitous Healthcare Service with Voice Inception Technique in Elderly Diabetic Patients

**DOI:** 10.1038/srep18214

**Published:** 2015-12-11

**Authors:** Kyoung Min Kim, Kyeong Seon Park, Hyun Ju Lee, Yun Hee Lee, Ji Seon Bae, Young Joon Lee, Sung Hee Choi, Hak Chul Jang, Soo Lim

**Affiliations:** 1 Department of Internal Medicine, Seoul National University Bundang Hospital and Seoul National University College of Medicine, Seongnam, 13620, South Korea; 2 u-healthcare center, Seoul National University Bundang Hospital and Seoul National University College of Medicine, Seongnam, Korea; 3 Aimmed Corporation Limited, Seoul, Korea

## Abstract

We have demonstrated previously that an individualized health management system using advanced medical information technology, named ubiquitous (u)-healthcare, was helpful in achieving better glycemic control than routine care. Recently, we generated a new u-healthcare system using a voice inception technique for elderly diabetic patients to communicate information about their glucose control, physical activity, and diet more easily. In a randomized clinical trial, 70 diabetic patients aged 60–85 years were assigned randomly to a standard care group or u-healthcare group for 6 months. The primary end points were the changes in glycated hemoglobin (HbA_1c_) and glucose fluctuation assessed by the mean amplitude glycemic excursion (MAGE). Changes in body weight, lifestyle, and knowledge about diabetes were also investigated. After 6 months, the HbA_1c_ levels decreased significantly in the u-healthcare group (from 8.6 ± 1.0% to 7.5 ± 0.6%) compared with the standard care group (from 8.7 ± 0.9% to 8.2 ± 1.1%, *P* < 0.01). The MAGE decreased more in the u-healthcare group than in the standard care group. Systolic blood pressure and body weight decreased and liver functions improved in the u-healthcare group, but not in the standard care group. The u-healthcare system with voice inception technique was effective in achieving glycemic control without hypoglycemia in elderly diabetic patients (Clinicaltrials.gov: NCT01891474).

Previous use of telemedicine has involved simple phone- or Internet-based systems in medical care^1^,^2^,^3^. The classic concept of telemedicine has been evolving to a ubiquitous (u)-healthcare system with the use of advanced information technologies and now includes close monitoring and instant feedback based on a clinical decision support system (CDSS), which operates regardless of time or place. Real-time individualized feedback to data transferred from monitoring devices is essential in current u-healthcare systems^4^,^5^,^6^.

Several studies have shown that applications of u-healthcare system help patients improve their glucose control and avoid critical events such as hypoglycemia or weight gain^7^,^8^. We previously shown the efficacy of a u-healthcare system with a sophisticated CDSS rule engine in helping patients achieve glucose control over 6 months^8^, and its effects were maintained for 1 year after the end of the original study^9^. However, the use of the monitoring device was revealed to be an important limitation particularly in elderly people. Introduction of simpler methods seemed to be crucial to the successful implementation of a u-healthcare system.

Today, the management of diabetes includes glucose control, regular exercise, and a healthy diet. However, many patients with diabetes undertake insufficient physical activity and have undesirable dietary habits^10^, and lifestyle intervention is most effective in delaying the progression of diabetes mellitus^11^,^12^. However, maintenance of a healthy lifestyle is difficult to follow without supervision^13^.

An individualized approach that focuses on not too strict blood glucose control and reducing the incidence of hypoglycemia has been emphasized recently in the management of patients with diabetes based on results obtained from large clinical trials^14^,^15^. This tailored approach is particularly needed for older adults with diabetes mellitus because hypoglycemia might do more harm in this group^16^. To achieve good glycemic control without hypoglycemia, consistent monitoring of glucose levels and instant individualized feedback must be provided in an easier way for the patient. Evaluation of daily dietary patterns and physical activity is also helpful for achieving better glucose control without causing hypoglycemia and weight gain. However, inputting information about diet on the Web site or mobile application was a major hurdle for the diet control in the u-healthcare service because of the diversity in the patients’ diets.

To solve this problem, we recently generated a new u-healthcare system using a voice inception technique for monitoring information about glucose control, physical activity, and diet. We believe that this new method is easier to use. We investigated the efficacy of the new u-healthcare service in managing glucose control without causing hypoglycemia in an elderly population.

## Materials and Methods

### Study Design and Participants

We undertook a randomized, controlled trial. Patients with type 2 diabetes mellitus, aged 60–85 years with a glycated hemoglobin (HbA_1c_) level of 7.0–11.0% (53–97 mmol/mol) were recruited from the outpatient clinic of the Seoul National University Bundang Hospital (SNUBH) from August 2013 to April 2014. There was no limitation on the use of oral antidiabetic agents in the enrollment. Patients with basal insulin and premixed insulin were also included.

The exclusion criteria were patients with type 1 diabetes or those needing multiple insulin injections; regular use of systemic corticosteroid within the preceding 6 months; a history of myocardial ischemia, heart failure of the New York Heart Association Class III-IV, thyroid disease with an abnormal thyroid function test, or severe liver or kidney disease; and the use of antiobesity drugs within the preceding 3 months. Because the use of a mobile device and the Internet was essential in this study, patients who were unable to use text messaging or to access the Internet were also excluded.

Based on our previous study^17^, for this study, we determined that the 95% confidence interval for a difference in HbA_1c_ levels after 6 months of intervention with an absolute change in the 0.54% HbA_1c_ level would be obtained using 70 patients, who would be assigned to either the u-healthcare group or the standard care group (α-error = 5%; β-error = 80%; assumed withdrawal rate = 10%).

A total of 90 patients were screened for the study and 20 were excluded (15 because of the exclusion criteria and five who refused to participate in the study after being informed of the study protocol). Finally, 70 patients were enrolled. The enrolled patients were randomly assigned to one of two groups using block randomization: 35 in the standard care group with self-monitored blood glucose and 35 in the u-healthcare group (Supplementary Fig. 1). The study protocol was registered at Clinicaltrials.gov (NCT01891474) on June 28th 2013. All procedures performed in this study were approved by the SNUBH institutional review board (B-1305/203-010) and all subjects were fully informed regarding study participation, and written informed consent was obtained from each individual (see the supplementary file for detailed study protocol). The study was performed in accordance with the Helsinki Declaration of 1975, as revised in 2008^18^.

### Procedures

For both groups, pertinent diabetes education including a therapeutic lifestyle change program was provided to standardize each patient’s understanding of diabetes management according to the recommendations of the American Diabetes Association (ADA) and the Korean Diabetes Association^19^,^20^.

All participants were instructed to measure their blood glucose levels at least seven times a week (three or more times fasting, three or more times postprandially, and once or more at bedtime). Each patient was advised to use their own glucometer.

At baseline, the doctors in the u-healthcare center assessed each patient’s glucose control status, average caloric intake, and the amount of regular physical activity. Based on these data, an individualized glycemic target goal was given to each participant. The patients in both groups were also equally educated about diet and exercise based on their previous dietary patterns and physical activity levels.

#### U-Healthcare System

**Figure 1:**
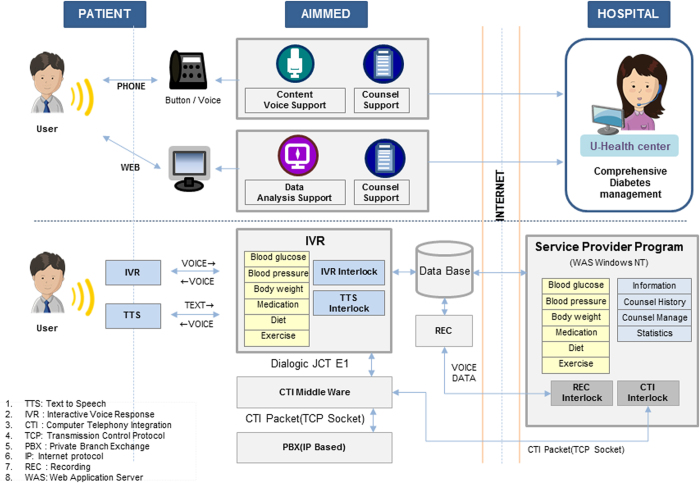
Scheme of the u-healthcare service using a voice inception IT solution. The participants in the u-healthcare group are educated about how to use the u-healthcare service. They are instructed to input their self-measured glucose levels into the u-healthcare service system using two alternative channels. (1) Channel 1 uses a mobile phone or landline, and it is possible to enter the health data through voice with ARS or touch pad tone. (2) Channel 2 uses the Web site in a conventional way, in which the user logs in to the disease-management Web site by typing the information directly into the patient’s administration page. Data input is automatically transmitted to the main server in the u-healthcare center and then tailored messages automatically generated from the CDSS rule engine are transmitted back to their phones instantly as a voice service or to their personal page on the Web site and stored in the u-healthcare system. For dietary feedback, participants are educated to give information on their food intake via phone. They are also able to upload a food diary or a picture of a food on the health data input page on the Web site. In the Web site, a list of each participant’s favorite food is uploaded by a dietician to help patient click on what and how much they eat. Nutritionists directly analyze the customer-specific dietary problems through the analysis program called CAN-Pro 4.0 (Korean nutrition Society). The results from the CAN-Pro software are displayed automatically on the Web site, and the participants can find detailed information about their dietary pattern or, if necessary, a nutritionist directly provides direct consultations via the phone.

Participants in the u-healthcare group were educated to use the u-healthcare service using the voice inception technique. They were guided to check their blood glucose levels at the same frequency as the standard care group. The scheme of the u-healthcare service used in this study is as follows (Fig. 1). Participants were asked to send their health data such as blood glucose level, body weight, exercise, diet, and medication adherence to the u-healthcare center through the auto response system (ARS) or touch pad system (text to speech, TTS) with a mobile phone or a landline. When the registered patients enter their health data using the ARS or touch pad system, it is recognized by the interactive voice response (IVR) system. The patients’ data are analyzed by the CDSS, which is determined by each patient’s clinical institution when he or she is registered at the study. Finally, the patients receive tailored feedback messages by voice or text messages, which are generated automatically through the CDSS rule engine for the data that they entered (Fig. 1). Alternatively, patients can also log in to the u-healthcare Web site (online u-healthcare center). When they enter their health data in their own page, they can receive tailored messages in the same way as the voice inception method.

For long-term management, an individualized health report is provided as an analysis of each individual’s cumulative data and corresponding messages about the management goal. Detailed information about diabetes management, recommended dietary pattern, and exercise type, and calorie consumption is provided on the online Web pages. Each person can also consult with healthcare professionals through an online bulletin board managed by the u-healthcare center. Comprehensive education for diabetes management was provided to all participants in face-to-face meetings at baseline and at each visit. In addition, detailed health-related information about diabetes mellitus and guidelines for diet and exercise helpful to the study participants were made available on the Web site. The patients can call the u-healthcare center when there is an emergency. The detailed protocol for the current study can be found in the supplement (**Supplementary study protocol**).

#### Dietary Intervention and Assessment

All subjects in this study were instructed to complete a 3-day food record (two weekdays and one day on a weekend) at the baseline visit and at the end of the study. Using proven food models, nutritionists evaluated the intakes and amounts of food consumed. At the baseline visit, the nutritionists provided 30–45 min of nutrition education including information about the components of a balanced diet, adequate caloric intake, and the importance of food choice to all participants. The nutritionists also advised overweight subjects to consume less carbohydrate and saturated fat, and encouraged underweight subjects to increase their protein intake and, for those with a high sodium intake, to lessen their sodium intake. During the study period, the participants received additional advice from the nutritionists, if needed, about modifying undesirable eating behaviors, such as inadequate dietary intake, late-night snacking, and binge eating. The 3-day food records were analyzed using the CAN-Pro 4.0, a computer-aided nutritional analysis program developed by the Korean Nutrition Society (Seoul, Korea)^21^.

#### Exercise Intervention and Assessment

The daily amount of exercise was assessed and the average caloric consumption by exercise was estimated at baseline in all participants. All subjects in this study were instructed to maintain or increase daily activity to more than 30 min at least three times a week. Patients in the u-healthcare group were guided to input the time and type of exercise every day by phone or the Web site. The caloric consumption was calculated at 3 and 6 months as follows: calories (kcal) = 1.05 × metabolic equivalents (METs) × body weight × time (h). The activity levels were calculated as 3.3 METs for light activity, 4 METs for moderate-intensity exercise, and 8 METs for vigorous exercise. The data were automatically sent back to the patient’s cell phone and Web site. In the standard care group, average caloric consumption by exercise was estimated at the 3- and 6-month visits.

#### Seven-point Self-monitoring of Blood Glucose (SMBG) and Assessment of Glucose Variability

The 7-point SMBG and mean amplitude glycemic excursion (MAGE) were used to assess glycemic variability. All participants were instructed to record a 7-point SMBG profile (preprandial and 2-h postprandial at each meal, and at bedtime) for 3 consecutive days at the baseline and end of the study period. MAGE was calculated by measuring the arithmetic mean of the differences between the peak and nadir values of blood glucose level.

#### Surveys of Health-related quality of life (HRQOL), Diabetes Self-management, and Knowledge of the management of Diabetes Mellitus

Three surveys were used to assess the changes of quality of life, and self-management and knowledge of diabetes mellitus after intervention: the health-related quality of life (HRQOL), diabetes self-management, and knowledge of the management of diabetes mellitus. All study participants completed these questionnaires at baseline and at the end of study period. To improve the accuracy of questionnaires, the research coordinator in the u-healthcare center asked questions and recorded participants’ answers in person.

#### Short Form 36 (SF-36)

To assess the HRQOL, the SF-36 questionnaire was used. The SF-36 is a short questionnaire with 36 items that measure eight variables; physical functioning, social functioning, role limitations due to physical problems, role limitations due to emotional problems, mental health, vitality, pain, and general perception of health^22^. The reliability and validity of the Korean version of the SF-36 has been reported^23^.

#### Summary of Diabetes Self-Care Activities (SDSCA)

To evaluate the level of diabetes self-management, the SDSCA questionnaire was used. The SDSCA is a questionnaire used to assess the level of self-care in adults with diabetes by measuring the frequency of performing diabetes self-care activities, including diet, exercise, blood glucose testing, foot care, and tobacco use, over the preceding 7 days^24^.

#### Michigan Diabetes Knowledge Test (MDKT)

Knowledge about diabetes was assessed using the MDKT, which comprises 14 multiple-choice questions about basic knowledge of diabetes and nine queries about knowledge of insulin use^25^.

### Study End Points

The primary end points of the present study were: (1) changes in HbA_1c_ levels and (2) glucose variability after the 6-month study period. A modified MAGE was used to assess glucose variability^26^.

The secondary end points were the percentage of patients achieving the target of HbA_1c_ ≤7.5% or ≤8.0% at 6 months. Hypoglycemia and adherence to medication were also evaluated during the study period. For both groups, the frequency of SMBG was checked to ensure compliance with self-testing. All participants visited the u-healthcare center every 3 months after randomization. The sulfonylurea or insulin dose could be adjusted downward for safety reason throughout the 6-month period.

### Definition of Hypoglycemia

All patients were educated on the symptoms of hypoglycemia in detail. They were advised to perform glucose testing immediately if they felt any hypoglycemic symptoms. In this study, hypoglycemia and severe hypoglycemia were defined as a blood glucose concentration <72 mg/dl (<4 mmol/l) with hypoglycemic symptoms and <50 mg/dl (<2.78 mmol/l) and requiring the assistance of another party, respectively.

### Statistical Analysis

All results are expressed as the mean ± SD. The Kolmogorov-Smirnov test was used to confirm the normal distribution of variables. The triglyceride concentrations were not normally distributed. Therefore, we used log-transformed values for triglyceride levels in the analyses. Considering the characteristics of the current study, per-protocol analysis was planned. Paired *t* tests were used to compare changes in parameters before and after intervention. Student’s *t* test and the χ^2^ test were used to compare differences between groups. Analyses were performed using SPSS Statistics for Windows (version 18.0; SPSS, Inc., Chicago, IL, USA). For all tests, *P* < 0.05 was considered statistically significant.

## Results

### Baseline Characteristics and Follow Up of Study Participants

There were no significant differences in baseline clinical and biochemical parameters including age, BMI, FPG, HbA_1c_ level, lipid profile, use of medication, lifestyles, or comorbidity between the u-healthcare group and the standard care group (Supplementary Table 1).

Thirty-three of the 35 patients enrolled in each group (94.3%) completed the study. One patient in the u-healthcare group did not use the u-healthcare system from the beginning, and one in the standard care group did not follow up. One patient from each group withdrew because an antidiabetic medication was added during the study period.

### Achievement of the Study End Points

#### Changes in HbA_1c_ Levels

**Figure 2:**
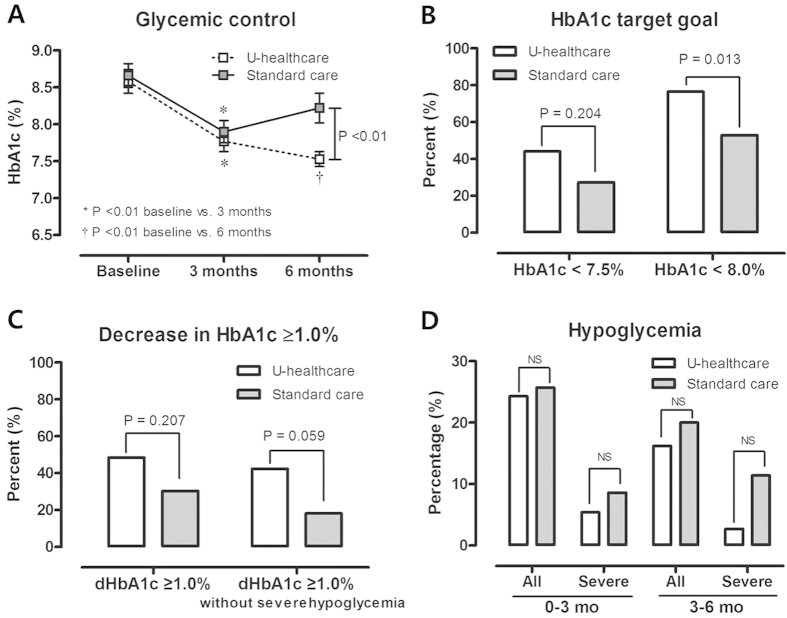
Comparison of glycemic control and hypoglycemia between the u-healthcare and standard care groups. (A) Changes in HbA_1c_ levels over the 6 months of the study in the u-healthcare and standard care groups. The data were expressed as the mean ± SD. (**B**) Percentages of patients who achieved the target level of HbA_1c_ ≤7.5% or ≤8.0% without hypoglycemia at 6 months. (**C**) Percentages of patients whose HbA_1c_ decreased by ≥1% with and without severe hypoglycemia during the study period. (**D**) Percentages of patients experiencing all and severe hypoglycemic events during the 3 and 6 months.

At the 3-month follow-up, the mean HbA_1c_ levels of the u-healthcare and standard care groups had decreased significantly from 8.6% to 7.8% and from 8.7% to 7.9%, respectively (both *P* < 0.01) (Fig. 2A). In the u-healthcare group, the HbA_1c_ level decreased further to 7.5% in the next 3 months, but that in the standard care group rebounded to 8.2% during this period. This resulted in a significant difference in the HbA_1c_ levels between the two groups at the end of the 6-month period of study (Fig. 2A). In the per-protocol analysis, the change in the HbA_1c_ level, the primary endpoint, was 0.96% in the u-healthcare group and 0.34% in the standard care group during the study period (*P* = 0.014).

Alternatively, ANCOVA was conducted to analyze the HbA_1c_ level at 6 months with the covariates of age, BMI, SBP, and baseline HbA1c level, serum creatinine, total cholesterol, and ALT. The adjusted HbA_1c_ level at the end of the study period was significantly lower in the u-healthcare group than in the standard care group (7.51 ± 0.16% in the u-healthcare group vs. 8.24 ± 0.16% in the standard care group, *P* = 0.002) (Table 1).

Twenty-seven of the 33 patients in the u-healthcare group used the u-healthcare service as instructed. Including only these compliant patients in the analysis of the primary end point increased the difference in the changes in HbA_1c_ between groups after 6 months (1.07% vs. 0.34%, *P* = 0.007). The other 6 (18.2%) patients had connected to the u-healthcare system primarily through the Web site. The major clinical outcomes did not differ between these two groups at the end of the study (Supplementary Fig. 2).

In the present study, only 3 participants consulted with their health professional through the online bulletin board. The low use of online bulletin board may be explained by the fact that all the study patients were intensively educated about their diet and exercise at baseline and 3-month visit, and that they had received tailored messages through the CDSS algorithm about their glucose levels, exercise, and diet.

#### Glucose Variability based on SMBG Profiles

**Figure 3:**
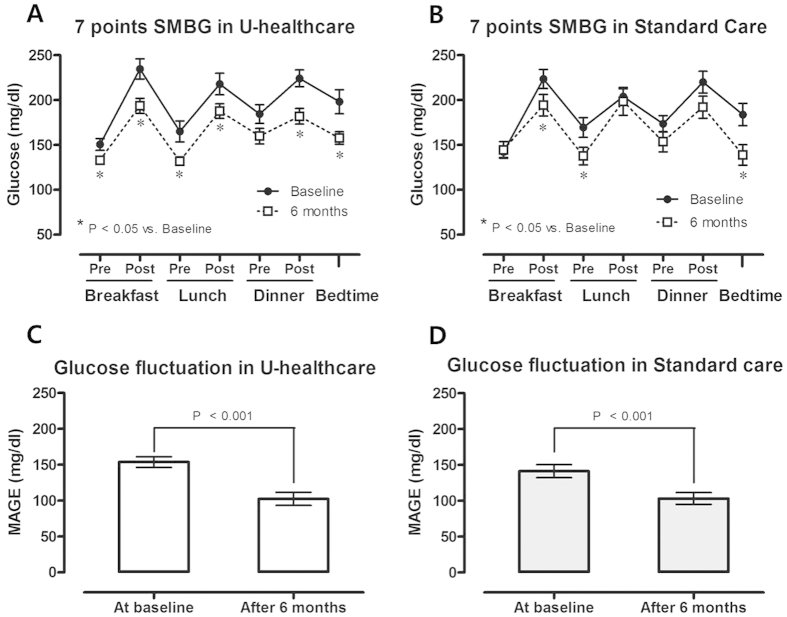
Seven-points SMBG for 3 days before and after the intervention (A,B) and glucose fluctuation assessed by the modified mean amplitude of glycemic excursion (MAGE) using the SMBG profiles. The data were expressed as the mean ± SD.

In the 7-points SMBG profiles, all values except that at pre-dinner time decreased significantly in the u-healthcare group, but only those at the postbreakfast, prelunch, and bedtime decreased significantly in the standard care group (Fig. 3A,B).

In the assessment of glucose variability, the MAGE using the SMBG profiles decreased significantly (153.9 ± 7.4 mg/dl to 102.5 ± 9.1 mg/dl and 141.3 ± 9.1 mg/dl to 103.2 ± 8.2 mg/dl in the u-healthcare and standard care groups, respectively; both *P* < 0.01 (Fig. 3C,D). However, there was no difference in the decrease in MAGE between the two groups.

#### Treatment Target Goal

The number of patients reaching the target HbA_1c_ levels of ≤7.5% and ≤8.0% after 6 months of follow-up was higher in the u-healthcare group than the standard care group (45.5% vs. 27.3% for ≤7.5%, *P* = 0.188 and 75.8% vs. 51.5% for ≤8.0%, *P* = 0.011, respectively). Importantly, the percentage of patients whose HbA_1c_ level decreased ≥1% without severe hypoglycemia after 6 months of follow-up tended to be higher in the u-healthcare group than the standard care group (42.4% vs. 18.2%; *P* = 0.059) (Fig. 2C).

#### Body Weight, BMI, and Waist Circumference

In the u-healthcare group, body weight, BMI, and waist circumference decreased significantly, whereas only waist circumference decreased in the standard care group. The changes in body weight and BMI in the u-healthcare group were significantly greater than those in the standard care group (Table 2).

#### Compliance

Adherence to medication during the study period did not differ between the two groups. For both groups, the frequency of SMBG was checked to ensure compliance of self-testing and did not differ between the two groups.

#### *Overall and Severe Hypoglycemia*

Before enrollment, the number of hypoglycemic events experienced by patients did not differ between the two groups. During the 6 months of the study, the number of patients who experienced hypoglycemia did not differ between groups, although severe hypoglycemia tended to occur more frequently in the standard care group than in the u-healthcare group (Fig. 2D).

#### Caloric Intake and Exercise

The mean caloric and carbohydrate intakes decreased slightly in both groups, but these changes were not significant. The sodium intake decreased significantly in patients in the u-healthcare group, but the decrease did not lead to a statistical difference between the study groups (Table 2). The caloric consumption from daily exercise increased significantly from around 250 kcal/day at the baseline to 486.2 kcal/day at 3 months and to 412.2 kcal/day at 6 months in the u-healthcare group, but not in the standard care group.

All study participants were allowed to receive advice from the nutritionists if needed during the study period. Additional nutritional advice was received an average of 3.48 times, and this value did not differ between the standard care group and the u-healthcare group. The clinical outcomes also did not differ between users of frequent nutritional support (≥2 times during the study period) and those with infrequent nutritional support (0 or 1 time).

#### SF-36, SDSCA, and MDKT Scores

There were no significant changes in SF-36, SDSCA, or MDKT scores during the study period in either study group (Table 2).

## Discussion

In this study, the patients who used the u-healthcare service with voice inception to communicate information about their glucose control, physical activity, and diet achieved improved glycemic control with less-frequent hypoglycemia. They also lost body weight, increased exercise, and reduced caloric intake compared with the standard care group. Thus, this user-friendly u-healthcare service led to decreased HbA_1c_ levels, the primary end point, in these elderly patients with diabetes.

The percentage of patients who achieved the target glycemic goal of HbA_1c_ ≤8.0% was greater in the u-healthcare group than in the standard care group. Considering the old age and high comorbidity in these patients, an HbA_1c_ of ≤8.0% seemed appropriate for their glucose control target according to the recent ADA guideline^20^. Hypoglycemia is now seen as one of the most dangerous factors and should be avoided in diabetes management because it might increase cardiovascular or all-cause mortality rates, particularly in older patients with diabetes^27^,^28^. Of note, elderly patients with diabetes tend to be less aware of hypoglycemic symptoms^29^. We also found that application of the u-healthcare system enabled more participants to decrease their HbA_1c_ levels by ≥1.0% without severe hypoglycemia compared with routine care, which is another important analysis point.

In this study, severe hypoglycemia seemed to occur less frequently in the u-healthcare group than in the standard care group although the numbers of overall hypoglycemic events did not differ significantly between groups. This finding corresponds with the result of our previous study showing less frequent severe or nocturnal hypoglycemia events in people who used an immediate feedback system for inputting glucose values compared with those who used self-monitoring of blood glucose levels^17^. Based on these two studies’ findings, it is conceivable that the u-healthcare service is effective in decreasing the frequency of hypoglycemic events by immediately alerting patients so that they can manage their hypoglycemia.

At every visit, the patients were educated about the importance of exercise and how to exercise was taught to every individual participant by an exercise specialist. Individual current dietary habits were assessed, and nutrition education was given to all patients by a dietician. During the study period, the HbA_1c_ levels decreased continuously in the u-healthcare group, whereas the HbA_1c_ levels decreased after 3 months and rebounded at 6 months in the standard group. This result implies that self-management of diabetes is not easy without supervision. Thus, immediate feedback on each patient’s glucose levels and timely recommendations seem to be more effective in managing diabetes than standard care with intensified SMBG alone.

In addition to better glycemic control, the u-healthcare group showed a decrease in body weight, waist circumference, systolic blood pressure, and liver enzyme activities compared with the standard care group. The instant feedback generated by the CDSS rule engine seemed to have contributed to these beneficial results by guiding patients toward a healthier dietary pattern and by encouraging them to exercise.

In the past decade, there have been substantial advances in information technology used in medicine. Several years ago, a ZigBee communication protocol was introduced for better diabetes management^30^. More recently, the combination of an Internet-based glucose control system with a mobile device has improved metabolic parameters in obese patients with type 2 diabetes^7^,^31^. Our group also found that a u-healthcare service that generates instant feedback using mobile systems in response to each patient’s glucose level improved the efficacy of glucose control^8^. However, buying a new device and becoming used to it appear to be major hurdles in the development of new telemedicine systems.

In the present study, we adopted a new voice inception technique that helped patients use the system more easily because they could use their own glucometers and phones without requiring any specific devices. All that was required was to make a phone call and report their glucose concentration and activity levels. Reporting their food consumption took some time, but the participants felt much at ease speaking on the phone than entering the information into the Web site or writing it in the diet notes. The number of calories consumed by physical activity was calculated automatically and the dietary pattern was analyzed comprehensively whenever patients sent their data to the u-healthcare center. Evaluation of the diet was found to help the patients maintain healthier dietary habits. Feedback about their activity informed them of their physical activity levels and helped them to increase the frequency and amount of exercise.

For safety reason, the hypoglycemic events were recorded meticulously by the investigators, and timely guidance was given to the patients to avoid critical hypoglycemic events. With this active surveillance strategy, the percentage of patients whose HbA_1c_ level decreased by ≥1% without hypoglycemia was twice as high in the u-healthcare group as in the standard group.

In the present study, we did not use a specific physical activity monitoring device or application. Wearing a new device or manipulating a new application can be a burden to elderly participants, and may be an economic burden in the future when put to practical use. To avoid these limitations, we generated a new u-healthcare system using a voice inception technique without requiring any specific devices or applications.

In the present study, we found improving trends in the scores for the SF-36, SDSCA, and MDKT in both groups, but these trends were not statistically significant. The six-month study period might not have been long enough to induce significant changes in these measures of quality of life and self-care activities^3^,^32^. Alternatively, the initial baseline levels of the study participants might have been high and further changes were therefore unlikely. From this perspective, further long-term studies that focus on the efficacy of telemedicine in changing quality of life and self-care activities are needed.

This study has some limitations. First, the 6-month study period might not be long enough to evaluate the long-term effects of the current u-healthcare system. Second, the study population was limited to individuals who were able to use a mobile phone and the Internet.

McMahon *et al.*, reported on the superior efficacy of a Web-based care program in controlling diabetes compared with usual care. The decrease in HbA_1c_ level was much larger in the persistent users compared with the intermittent users among the Web-based care patients^33^. Other clinical outcomes were also dependent on the patients’ compliance with telemonitoring. In the present study, 82% of patients in the u-healthcare group (27/33) showed good compliance in using the u-healthcare system. This high compliance rate indicates that elderly patients prefer a simpler and easy way to use familiar devices to sophisticated or complicated ways requiring high-end technology. Thus, using the simple technology with voice inception technology has shown promise for improving the self-management of diabetes.

In conclusion, our study has demonstrated that the application of this simple u-healthcare service with voice reception technology enables elderly patients with diabetes to achieve better glycemic control without hypoglycemia and a healthier lifestyle compared with conventional care. We anticipate that this simple and easily applicable u-healthcare system will be useful in the management of diabetes in elderly people.

Of course, this system needs to be evaluated along multiple dimensions: long-term compliance, cost-effectiveness, and motivation for self-management. Assessment of the impact of this system on hard end points such as diabetes-related morbidity and mortality is also required.

## Additional Information

**How to cite this article**: Kim, K. M. *et al.* Efficacy of a New Medical Information system, Ubiquitous Healthcare Service with Voice Inception Technique in Elderly Diabetic Patients. *Sci. Rep.* **5**, 18214; doi: 10.1038/srep18214 (2015).

## Supplementary Material

Supplementary Information

## Figures and Tables

**Table 1:** Comparison of HbA_1c_ level (%) between the u-healthcare group and standard care group at 6-months.

	**U-healthcare group**	**Standard care group**	***P***†
	**(n = 33)**	**(n = 33)**	
HbA_1c_ (%)* at 6 months	7.51 ± 0.16	8.24 ± 0.16	0.002

^*^Adjusted mean ± standard error.

^†^*P*-value for the comparison between the 2 groups by ANCOVA adjusted for age, body mass index, systolic blood pressure, and the baseline levels of HbA_1c_, serum creatinine, total cholesterol, and alanine aminotransferase.

**Table 2:** Changes in anthropometrics, biochemical parameters, and frequency of self-monitoring blood glucose by both groups after 6 months.

	**U-healthcare group (n = 33)**		**Standard care group (n = 33)**		***P***†
	**Baseline**	**6 months**	**Baseline**	**6 months**	
	**mean** ± **SD**	**Mean** ± **SD**	**Mean** ± **SD**	**Mean** ± **SD**	
Weight, kg	64.9 ± 12.0	63.5 ± 12.6*	65.9 ± 10.1	65.5 ± 10.2	0.035
BMI, kg/m^2^	25.2 ± 3.4	24.5 ± 3.5*	25.1 ± 2.9	25.0 ± 3.0	0.027
Waist circ., cm	89.1 ± 9.0	86.8 ± 9.2*	89.8 ± 7.2	88.4 ± 7.1*	NS
SBP, mmHg	132.2 ± 9.4	123.9 ± 13.5*	133.1 ± 13.3	126.8 ± 11.6*	NS
DBP, mmHg	75.0 ± 6.3	71.4 ± 8.0*	75.5 ± 6.3	73.1 ± 6.9	NS
FPG, mg/dl	173.0 ± 49.1	139.0 ± 25.9*	150.8 ± 49.6	148.3 ± 47.3	0.012
HbA_1c_, %	8.5 ± 0.8	7.5 ± 0.6*	8.6 ± 0.9	8.2 ± 1.1	0.014
HbA_1c_, mmol/mol	69.3 ± 8.7	58.8 ± 6.4	70.1 ± 9.7	66.4 ± 12.5	0.014
Cr, mg/dl	0.86 ± 0.30	0.83 ± 0.27	0.85 ± 0.260	87 ± 0.30	NS
eGFR	84.7 ± 26.9	85.8 ± 22.9	84.6 ± 22.2	84.1 ± 23.1	NS
Total C, mg/dl	166.8 ± 33.1	162.9 ± 38.6	158.5 ± 29.0	154.5 ± 32.2	NS
TG‡, mg/dl	151.5 ± 82.8	137.8 ± 71.1	145.1 ± 104.6	138.7 ± 69.9	NS
HDL-C, mg/dl	51.0 ± 14.8	52.3 ± 16.6	49.5 ± 11.6	47.2 ± 8.8	NS
LDL-C, mg/dl	87.2 ± 28.5	88.4 ± 24.2	84.6 ± 24.7	80.7 ± 26.6	NS
AST, IU/l	27.8 ± 17.5	24.5 ± 6.3	23.1 ± 8.0	22.5 ± 11.6	NS
ALT, IU/l	32.7 ± 20.8	25.9 ± 10.5*	22.5 ± 10.3	19.5 ± 10.5*	NS
***Survey***					
SF-36	520.0 ± 114.5	544.8 ± 142.1	534.9 ± 152.2	533.8 ± 144.9	NS
SDSCA	23.6 ± 4.1	26.9 ± 3.4	23.2 ± 5.0	27.1 ± 4.0	NS
MDKT	64.9 ± 11.7	67.3 ± 11.3	61.8 ± 14.4	63.1 ± 11.7	NS
***Diet***					
Total calorie, kcal/day	1646.4 ± 614.0	1566.8 ± 294.1	1623.3 ± 332.2	1511.9 ± 329.8	NS
Carbohydrate, g	253.5 ± 50.3	238.8 ± 51.8	268.4 ± 62.1	244.4 ± 4.8	NS
Protein, g	70.9 ± 18.7	71.2 ± 17.5	66.0 ± 17.2	65.0 ± 17.4	NS
Fat, g	40.5 ± 12.5	40.2 ± 11.5	34.5 ± 13.5	35.1 ± 12.0	NS
Sodium, mg	5054.4 ± 2214.8	4092.5 ± 1576.8*	4881.8 ± 1819.1	4550.5 ± 1863.7	NS

BMI, body mass index; SBP, systolic blood pressure; DBP, diastolic blood pressure; FPG, fasting plasma glucose; Cr, creatinine; eGFR, estimated glomerular filtration rate; C, cholesterol; TG, triglyceride, AST, aspartate aminotransferase; ALT, alanine aminotransferase; HDL, high-density lipoprotein; LDL, low-density lipoprotein.

^*^P < 0.05 from Paired *t* tests used to compare changes in parameters before and after intervention.

^†^*P*-value for the comparison of the changes in each variable between the 2 groups using Student’s *t* test.

^‡^Log-transformed values were used for comparison.

## References

[b1] Ding H., Moodley Y., Kanagasingam Y. & Karunanithi M. A mobile-health system to manage chronic obstructive pulmonary disease patients at home. Conf Proc IEEE Eng Med Biol Soc 2012, 2178–2181 (2012). 23366354 10.1109/EMBC.2012.6346393

[b2] Steventon A. *et al.* Effect of telehealth on use of secondary care and mortality: findings from the Whole System Demonstrator cluster randomised trial. BMJ 344, e3874 (2012). 22723612 10.1136/bmj.e3874 PMC3381047

[b3] de Jongh T. *et al.* Mobile phone messaging for facilitating self-management of long-term illnesses. Cochrane Database Syst Rev 12, Cd007459 (2012). 23235644 10.1002/14651858.CD007459.pub2 PMC6486189

[b4] Alasaarela E. & Oliver N. S. Wireless solutions for managing diabetes: A review and future prospects. Technol Health Care 17, 353–367 (2009). 20051615 10.3233/THC-2009-0554

[b5] Perez-Ferre N. *et al.* A Telemedicine system based on Internet and short message service as a new approach in the follow-up of patients with gestational diabetes. Diabetes Res Clin Pract 87, e15–17 (2010). 20044162 10.1016/j.diabres.2009.12.002

[b6] Mulvaney S. A. *et al.* An internet-based program to improve self-management in adolescents with type 1 diabetes. Diabetes Care 33, 602–604 (2010). 20032275 10.2337/dc09-1881 PMC2827516

[b7] Cho J. H. *et al.* Long-term effect of the Internet-based glucose monitoring system on HbA1c reduction and glucose stability: a 30-month follow-up study for diabetes management with a ubiquitous medical care system. Diabetes Care 29, 2625–2631 (2006). 17130195 10.2337/dc05-2371

[b8] Lim S. *et al.* Improved glycemic control without hypoglycemia in elderly diabetic patients using the ubiquitous healthcare service, a new medical information system. Diabetes Care 34, 308–313 (2011). 21270188 10.2337/dc10-1447 PMC3024339

[b9] Kang S. M. *et al.* Ubiquitous healthcare service has the persistent benefit on glycemic control and body weight in older adults with diabetes. Diabetes Care 35, e19 (2012). 22355022 10.2337/dc11-2138 PMC3322707

[b10] Hu F. B. *et al.* Diet, lifestyle, and the risk of type 2 diabetes mellitus in women. N Engl J Med 345, 790–797 (2001). 11556298 10.1056/NEJMoa010492

[b11] Knowler W. C. *et al.* Reduction in the incidence of type 2 diabetes with lifestyle intervention or metformin. N Engl J Med 346, 393–403 (2002). 11832527 10.1056/NEJMoa012512 PMC1370926

[b12] Li G. *et al.* The long-term effect of lifestyle interventions to prevent diabetes in the China Da Qing Diabetes Prevention Study: a 20-year follow-up study. Lancet 371, 1783–1789 (2008). 18502303 10.1016/S0140-6736(08)60766-7

[b13] Wing R. R. *et al.* Cardiovascular effects of intensive lifestyle intervention in type 2 diabetes. N Engl J Med 369, 145–154 (2013). 23796131 10.1056/NEJMoa1212914 PMC3791615

[b14] Gerstein H. C. *et al.* Effects of intensive glucose lowering in type 2 diabetes. N Engl J Med 358, 2545–2559 (2008). 18539917 10.1056/NEJMoa0802743 PMC4551392

[b15] Patel A. *et al.* Intensive blood glucose control and vascular outcomes in patients with type 2 diabetes. N Engl J Med 358, 2560–2572 (2008). 18539916 10.1056/NEJMoa0802987

[b16] Munshi M. N., Maguchi M. & Segal A. R. Treatment of type 2 diabetes in the elderly. Curr Diab Rep 12, 239–245 (2012). 22484911 10.1007/s11892-012-0269-4

[b17] Lim S. *et al.* Multifactorial intervention in diabetes care using real-time monitoring and tailored feedback in type 2 diabetes. Acta Diabetol, 10.1007/s00592-015-0754-8. (2015). 25936739

[b18] World Medical Association declaration of Helsinki. Recommendations guiding physicians in biomedical research involving human subjects. JAMA 277, 925–926 (1997). 9062334

[b19] Ko S.-H. *et al.* 2011 Clinical practice guidelines for type 2 diabetes in Korea. Diabetes & Metabolism Journal 35, 431–436 (2011). 22111032 10.4093/dmj.2011.35.5.431 PMC3221016

[b20] Standards of medical care in diabetes–2014. *Diabetes Care* **37** Suppl 1, S14-80 (2014). 10.2337/dc14-S014 24357209

[b21] Cui H. S. *et al.* Dietary pattern and nutrient intake of korean children with atopic dermatitis. Ann Dermatol 26, 570–575 (2014). 25324648 10.5021/ad.2014.26.5.570 PMC4198583

[b22] Huang I. C. *et al.* Diabetes-specific or generic measures for health-related quality of life? Evidence from psychometric validation of the D-39 and SF-36. Value Health 11, 450–461 (2008). 18489668 10.1111/j.1524-4733.2007.00261.x

[b23] Han C. W. *et al.* Development of the Korean version of Short-Form 36-Item Health Survey: health related QOL of healthy elderly people and elderly patients in Korea. Tohoku J Exp Med 203, 189–194 (2004). 15240928 10.1620/tjem.203.189

[b24] Toobert D. J., Hampson S. E. & Glasgow R. E. The summary of diabetes self-care activities measure: results from 7 studies and a revised scale. Diabetes Care 23, 943–950 (2000). 10895844 10.2337/diacare.23.7.943

[b25] Fitzgerald J. T. *et al.* The reliability and validity of a brief diabetes knowledge test. Diabetes Care 21, 706–710 (1998). 9589228 10.2337/diacare.21.5.706

[b26] Service F. J. *et al.* Mean amplitude of glycemic excursions, a measure of diabetic instability. Diabetes 19, 644–655 (1970). 5469118 10.2337/diab.19.9.644

[b27] Snell-Bergeon J. K. & Wadwa R. P. Hypoglycemia, diabetes, and cardiovascular disease. Diabetes Technol Ther 14 Suppl 1, S51–58 (2012). 22650225 10.1089/dia.2012.0031 PMC3361183

[b28] McCoy R. G. *et al.* Increased mortality of patients with diabetes reporting severe hypoglycemia. Diabetes Care 35, 1897–1901 (2012). 22699297 10.2337/dc11-2054 PMC3425008

[b29] Bremer J. P. *et al.* Hypoglycemia unawareness in older compared with middle-aged patients with type 2 diabetes. Diabetes Care 32, 1513–1517 (2009). 19487634 10.2337/dc09-0114 PMC2713637

[b30] Lee H. J. *et al.* Ubiquitous healthcare service using Zigbee and mobile phone for elderly patients. Int J Med Inform 78, 193–198 (2009). 18760959 10.1016/j.ijmedinf.2008.07.005

[b31] Yoo H. J. *et al.* A Ubiquitous Chronic Disease Care system using cellular phones and the internet. Diabet Med 26, 628–635 (2009). 19538239 10.1111/j.1464-5491.2009.02732.x

[b32] Kim Y. J. *et al.* A Smartphone Application Significantly Improved Diabetes Self-Care Activities with High User Satisfaction. Diabetes Metab J 39, 207–217 (2015). 26124991 10.4093/dmj.2015.39.3.207 PMC4483606

[b33] McMahon G. T. *et al.* Web-based care management in patients with poorly controlled diabetes. Diabetes Care 28, 1624–1629 (2005). 15983311 10.2337/diacare.28.7.1624 PMC1262644

