# Supplementary material for: Efficacy of a New Medical Information system, Ubiquitous Healthcare Service with Voice Inception Technique in Elderly Diabetic Patients
Source: Sci Rep. 2015 Dec 11;5:18214. doi: 10.1038/srep18214 (PMC4676004; doi:10.1038/srep18214)
Supplement: Supplementary Information [file srep18214-s1.doc]

**Efficacy of a New Medical Information system, Ubiquitous Healthcare Service with Voice Inception Technique in Elderly Diabetic Patients**

**Running title: Voice Inception Technique in Diabetes Care**

Kyoung Min Kim^1,2^, Kyeong Seon Park^1,2^, Hyun Ju Lee^2^, Yun Hee Lee^2^, Ji Seon Bae^3^, Young Joon Lee^3^, Sung Hee Choi^1^, Hak Chul Jang^1^ & Soo Lim^1,2^

^1^Department of Internal Medicine, ^2^u-healthcare center, Seoul National University Bundang Hospital and Seoul National University College of Medicine, Seongnam, Korea

^3^Aimmed Corporation Limited, Seoul, Korea

**Corresponding author: Soo Lim, MD, PhD

Associate professor and chairperson of u-healthcare taskforce

Department of Internal Medicine, Seoul National University College of Medicine and Seoul National University Bundang Hospital

300, Gumi-dong, Bundang-gu, Seongnam-city, Korea (postal code: 463-707)

E-mail: [limsoo@snu.ac.kr](mailto:limsoo@snu.ac.kr) / Tel: 82-31-787-7035 / Fax: 82-31-787-4051

| **Supplementary Table 1—Baseline characteristics of participants in both groups** | | | | | | |
| --- | --- | --- | --- | --- | --- | --- |
|  | **U-healthcare group**  **(n = 35)** | | | **Standard care group**  **(n = 35)** | | |
| Male/female, n | 17/18 | | | 18/17 | | |
| Age, years | 65.7 | ± | 5.0 | 65.9 | ± | 5.7 |
| Duration of diabetes, years | 16.6 | ± | 10.5 | 14.6 | ± | 7.3 |
| Height, cm | 160.7 | ± | 8.7 | 162.5 | ± | 8.3 |
| Weight, kg | 65.2 | ± | 11.9 | 67.4 | ± | 11.6 |
| Body mass index, kg/m^2^ | 25.1 | ± | 3.3 | 25.4 | ± | 3.1 |
| Waist circumference, cm | 89.1 | ± | 8.8 | 90.6 | ± | 7.9 |
| Systolic blood pressure, mmHg | 132.2 | ± | 9.2 | 132.8 | ± | 13.1 |
| Diastolic blood pressure, mmHg | 74.7 | ± | 6.3 | 75.9 | ± | 6.4 |
| Fasting plasma glucose, mg/dl | 169.7 | ± | 49.6 | 155.0 | ± | 51.2 |
| HbA_1c_, % | 8.6 | ± | 1.0 | 8.7 | ± | 0.9 |
| HbA_1c_, mmol/mol | 70.2 | ± | 10.6 | 71.1 | ± | 10.3 |
| Creatinine, mg/dl | 0.89 | ± | 0.32 | 0.85 | ± | 0.25 |
| eGFR | 82.4 | ± | 27.8 | 85.0 | ± | 21.7 |
| Total cholesterol, mg/dl | 166.5 | ± | 32.2 | 159.4 | ± | 28.4 |
| Triglyceride, mg/dl | 159.1 | ± | 90.6 | 143.8 | ± | 103.3 |
| HDL-cholesterol, mg/dl | 50.4 | ± | 14.6 | 49.5 | ± | 11.4 |
| LDL-cholesterol, mg/dl | 87.5 | ± | 27.7 | 85.2 | ± | 24.5 |
| AST, IU/l | 27.3 | ± | 17.1 | 23.1 | ± | 7.7 |
| ALT, IU/l | 32.2 | ± | 20.5 | 23.3 | ± | 10.4 |
| *Medication for glucose control* |  | | |  | | |
| Sulfonylurea, N (%) | 21 (60.0) | | | 28 (80.0) | | |
| Metformin, N (%) | 30 (85.7) | | | 35 (100.0) | | |
| Thiazolidinedione, N (%) | 1 (2.9) | | | 3 (8.6) | | |
| DPP4 inhibitor, N (%) | 19 (54.3) | | | 15 (42.9) | | |
| α-glucosidase inhibitor, N (%) | 1 (2.9) | | | 2 (5.7) | | |
| Insulin, N (%) | 15 (42.9) | | | 10 (28.6) | | |
| *Antihypertensive medication* |  | | |  | | |
| RAS blockades, N (%) | 19 (54.3) | | | 21 (60.0) | | |
| Calcium channel blockers, N (%) | 14 (40.0) | | | 14 (40.0) | | |
| Diuretics, N (%) | 7 (20.0) | | | 4 (11.4) | | |
| Beta-blockers, N (%) | 5 (14.3) | | | 3 (8.6) | | |
| *Lipid-lowering medication* |  | | |  | | |
| Statins, N (%) | 25 (71.4) | | | 23 (65.7) | | |
| Other lipid lowering agents, N (%) | 8 (22.9) | | | 5 (14.3) | | |
| *Lifestyle* |  | | |  | | |
| Current drinker, N (%) | 9 (25.7) | | | 12 (34.3) | | |
| Current smoker, N (%) | 7 (20.0) | | | 5 (14.3) | | |
| Regular exercise, N (%) | 16 (45.7) | | | 16 (45.7) | | |
| *Comorbidity* |  | | |  | | |
| Hypertension, N (%) | 16 (45.7) | | | 18 (51.4) | | |
| Dyslipidemia, N (%) | 25 (71.4) | | | 23 (65.7) | | |
| Cardiovascular disease, N (%) | 8 (22.9) | | | 5 (14.3) | | |
| Retinopathy, N (%) | 2 (5.7) | | | 2 (5.7) | | |
| Data are presented as the mean ± SD or number of participants (%). eGFR, estimate glomerular filtration rate; HDL, high-density lipoprotein; LDL, low-density lipoprotein; AST, aspartate aminotransferase; ALT, alanine aminotransferase; DPP4, dipeptidyl peptidase 4; RAS, renin-angiotensin system. | | | | | | |

**Supplementary Fig. 1—CONSORT Flow Diagram**

**Assessed for eligibility (n = 90**)

**Excluded (n = 20)**

Not meeting inclusion criteria (n = 15)

Declined to participate (n = 5)

**Analysed (n = 33)**

Excluded from analysis (n = 0)

**Lost to follow-up (n = 2)**

Discontinued intervention (n = 1)

Other antidiabetic drug added (n = 1)

**Allocated to intervention A (n = 35)**

Received allocated intervention (n = 35)

Did not receive allocated intervention (n = 0)

**Lost to follow-up (n = 2)**

No show (n = 1)

Other antidiabetic drug added (n = 1)

**Allocated to intervention B (n = 35)**

Received allocated intervention (n = 35)

Did not receive allocated intervention (n = 0)

**Analysed (n = 33)**

Excluded from analysis (n = 0)

**Allocation**

**Analysis**

**Follow-Up**

**Randomized (n = 70)**

**Enrollment**

**Supplementary Fig. 2**—Comparison of HbA_1c_ levels and glycemic target goal without hypoglycemia between the phone users and website users. A. Changes in HbA_1c_ levels over the 6 months of the study in the phone users and website users. Values represent mean ± SD. B. Percentages of patients who achieved the target level of HbA_1c_ ≤7.5% or ≤8.0% without hypoglycemia at 6 months.


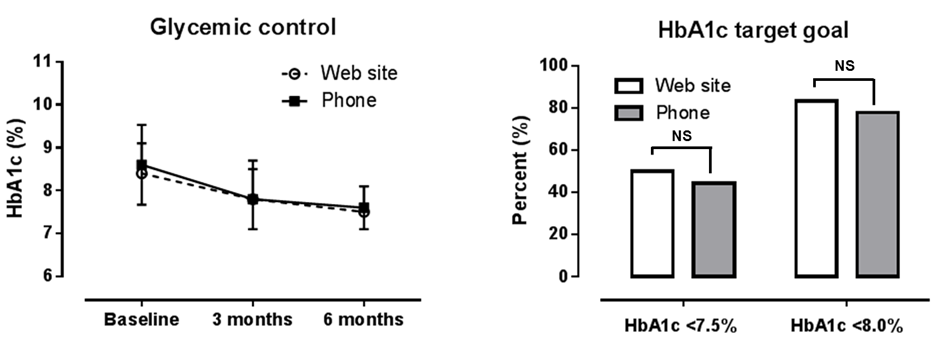


**A**

**B**

**Study Protocol**

**Efficacy of a New Medical Information system,** Ubiquitous Healthcare Service with Voice Inception Technique in Elderly Patients With Type 2 Diabetes Mellitus

|  | **Doc. No.: U13-2072-05** |
| --- | --- |
| **Title:** | Efficacy of a New Medical Information system, Ubiquitous Healthcare Service with Voice Inception Technique in Elderly Patients With Type 2 Diabetes Mellitus |
| **Primary investigator:** | Soo Lim, MD, PhD |
|  | Associate Professor |
|  | Department of Internal Medicine, Seoul National University College of Medicine and Seoul National University Bundang Hospital |
|  | 300, Gumi-dong, Bundang-gu, Seongnam-city, Korea (postal code: 463-707) |
|  | E-mail: limsoo@snu.ac.kr  Tel: 82-31-787-7035 / Fax: 82-31-787-4051 |
|  |  |
| **Co-investigator:** | Kyoung Min Kim, MD, PhD |
|  | Assistant Professor |
|  | Department of Internal Medicine, Seoul National University College of Medicine and Seoul National University Bundang Hospital |
|  | 300, Gumi-dong, Bundang-gu, Seongnam-city, Korea (postal code: 463-707) |
|  | E-mail: kyoungmin02@gmail.com  Tel: 82-31-787-7047 / Fax: 82-31-787-4051 |
|  |  |
| **Status:** | **Final Protocol** |
| **Version and Date:** | **Version: 1 Date: 13-DEC-2014** |

**Title of trial:** Efficacy of a New Medical Information system, Ubiquitous Healthcare Service with Voice Inception Technique in Elderly Patients With Type 2 Diabetes Mellitus

**Primary investigator:** Soo Lim, MD, PhD

Associate Professor

Department of Internal Medicine, Seoul National University College of Medicine and Seoul National University Bundang Hospital

300, Gumi-dong, Bundang-gu, Seongnam-city, Korea (postal code: 463-707)

E-mail: limsoo@snu.ac.kr Tel: 82-31-787-7035 / Fax: 82-31-787-4051

ABBREVIATIONS

| U-healthcare | Ubiquitous healthcare |
| --- | --- |
| CDSS | Clinical decision support system |
| SBP | Systolic blood pressure |
| DBP | Diastolic blood pressure |
| HbA1c | Glycated hemoglobin |
| GCP | Good Clinical Practice |
| EMR | Electronic medical record |
| ADA | American Diabetes Association |
| KDA | Korean Diabetes Association |
| NGSP | Glycohemoglobin Standardization Program |
| SOP | Standard operating procedure |
| SNUBH | Seoul National University Bundang Hospital |

**1. INTRODUCTION**

Previous use of telemedicine has involved simple phone- or Internet-based systems in medical care (1-3). The classic concept of telemedicine has been evolving to a ubiquitous (u)-healthcare system with the use of advanced information technologies and now includes close monitoring and instant feedback based on a clinical decision support system (CDSS), which operates regardless of time or place. Real-time individualized feedback to data transferred from monitoring devices is essential in current u-healthcare systems (4-6)

Several studies have shown that applications of u-healthcare system help patients improve their glucose control and avoid critical events such as hypoglycemia or weight gain (7,8). We previously shown the efficacy of a u-healthcare system with a sophisticated CDSS rule engine in helping patients achieve glucose control over 6 months (9), and its effects were maintained for 1 year after the end of the original study (10). However, the use of the monitoring device was revealed to be an important limitation particularly in elderly people. Introduction of simpler methods seemed to be crucial to the successful implementation of a u-healthcare system.

Today, the management of diabetes includes glucose control, regular exercise, and a healthy diet. However, many patients with diabetes undertake insufficient physical activity and have undesirable dietary habits (11), and lifestyle intervention is most effective in delaying the progression of diabetes mellitus (12,13). However, maintenance of a healthy lifestyle is difficult to follow without supervision (14).

An individualized approach that focuses on not too strict blood glucose control and reducing the incidence of hypoglycemia has been emphasized recently in the management of patients with diabetes based on results obtained from large clinical trials (15,16). This tailored approach is particularly needed for older adults with diabetes mellitus because hypoglycemia might do more harm in this group (17). To achieve good glycemic control without hypoglycemia, consistent monitoring of glucose levels and instant individualized feedback must be provided in an easier way for the patient. Evaluation of daily dietary patterns and physical activity is also helpful for achieving better glucose control without causing hypoglycemia and weight gain. However, inputting information about diet on the Web site or mobile application was a major hurdle for the diet control in the u-healthcare service because of the diversity in the patients’ diets.

To solve this problem, a new u-healthcare system is generated using a voice inception technique for monitoring information about glucose control, physical activity, and diet. We believe that this new method is easier to use. We will investigate the efficacy of the new u-healthcare service in managing glucose control without causing hypoglycemia in an elderly population.

**2. OBJECTIVE**

We have demonstrated previously that an individualized health management system using advanced medical information technology, named ubiquitous (u)-healthcare, was helpful in achieving glycemic control without hypoglycemia. Recently, we generated a new u-healthcare system using a voice inception technique to send tailored medical advice on the patients’ glucose levels, physical activity, and diet.

The goal of this study is to investigate efficacy of this new u-healthcare system using a voice inception technique in elderly patients with type 2 diabetes mellitus (T2DM).

**3. SUBJECTS and METHODS**

**[1] Study participants**

- **Inclusion criteria:**
- Patients with T2DM prior to informed consent
- Aged 60-85 years
- Glycated hemoglobin (HbA1c) levels: 7.0‒11.0% at baseline 1
- Signed and dated written informed consent by date of Visit 1 in accordance with Good Clinical Practice (GCP) and local legislation
- **Exclusion criteria:**
- Patients who are unable to use text messages or to access the internet. Current treatment with systemic steroids (orally taken or parenteral) at time of informed consent or change in dosage of thyroid hormones within 6 weeks prior to informed consent or any other uncontrolled endocrine disorder except T2DM
- Alcohol or drug abuse within the 3 months prior to informed consent that would interfere with trial participation or any ongoing condition leading to a decreased compliance to trial procedures in the judgment of the investigator
- Intake of an investigational drug in another trial within 30 days prior to intake of study medication in this trial or participation in the follow-up period of another trial (participation in observational studies is permitted)
- Any other clinical condition that, in the opinion of the investigator, would jeopardize patient’s safety while participating in this clinical trial
- **Removal of patients from trial**

An individual patient is to be withdrawn from the trial if:

• The patient withdraws consent, without the need to justify the decision.

• The patient is no longer able to participate for other medical reasons (e.g. surgery, AEs, or other diseases).

• Recurrent occurrence of hypoglycaemia that may put the patient at risk with continued participation.

**[2] Selection of subjects**

A sufficient number of patients will be screened for the trial to ensure that 60 patients are randomized to the trial's double-blind treatment period (30 patients in each treatment arm: u-healthcare group and standard care group). The screening failure rate for this trial is expected to be around 15% of patients. This rate would give an estimate of the number of patients needed to be screened to be 72.

**[3] Study endpoint**

- **Primary endpoint:**

1) Changes of HbA1c after 6 months of intervention

2) Glucose Variability assessed by 7 points SMBG for 3 days

- **Secondary endpoints:**
  - Target goal of HbA1c (≤7.5%) or HbA1c (≤8.0%)
  - Hypoglycemia
  - Drug adherence
  - Self-monitoring blood glucose compliance
  - Weight change
  - Quality of life assessed by SF36
  - Diabetes Self-Care Activities assessed by SDSCA
  - Michigan Diabetes Knowledge Test (MDKT)

**[4] Study Design and Procedures**

**1. Study groups**

Enrolled patients will be randomly assigned to one of two groups using block randomization **(Figure 1)**: 35 in the (1) standard care group with self-monitored blood glucose and 35 in the (2) u-healthcare group. For both groups, pertinent diabetes education including a therapeutic lifestyle change program are provided to standardize each patient’s understanding of diabetes management according to the recommendations of the American Diabetes Association (ADA) and the Korean Diabetes Association {Korean Diabetes Association, 2007 181 /id;American Diabetes Association, 2010 193 /id}. Before the beginning of each trial, patient in both groups receive diet and exercise counselling from a nutritionist. The counselling is based on local diet recommendations and should include a food log/intake booklet. The food intake booklets are provided as a tool. Record of the actual food intake over a time of three consecutive days before the clinic visit is requested at regular intervals. The patients are reminded to follow the agreed diet and exercise plan at every visit.

**
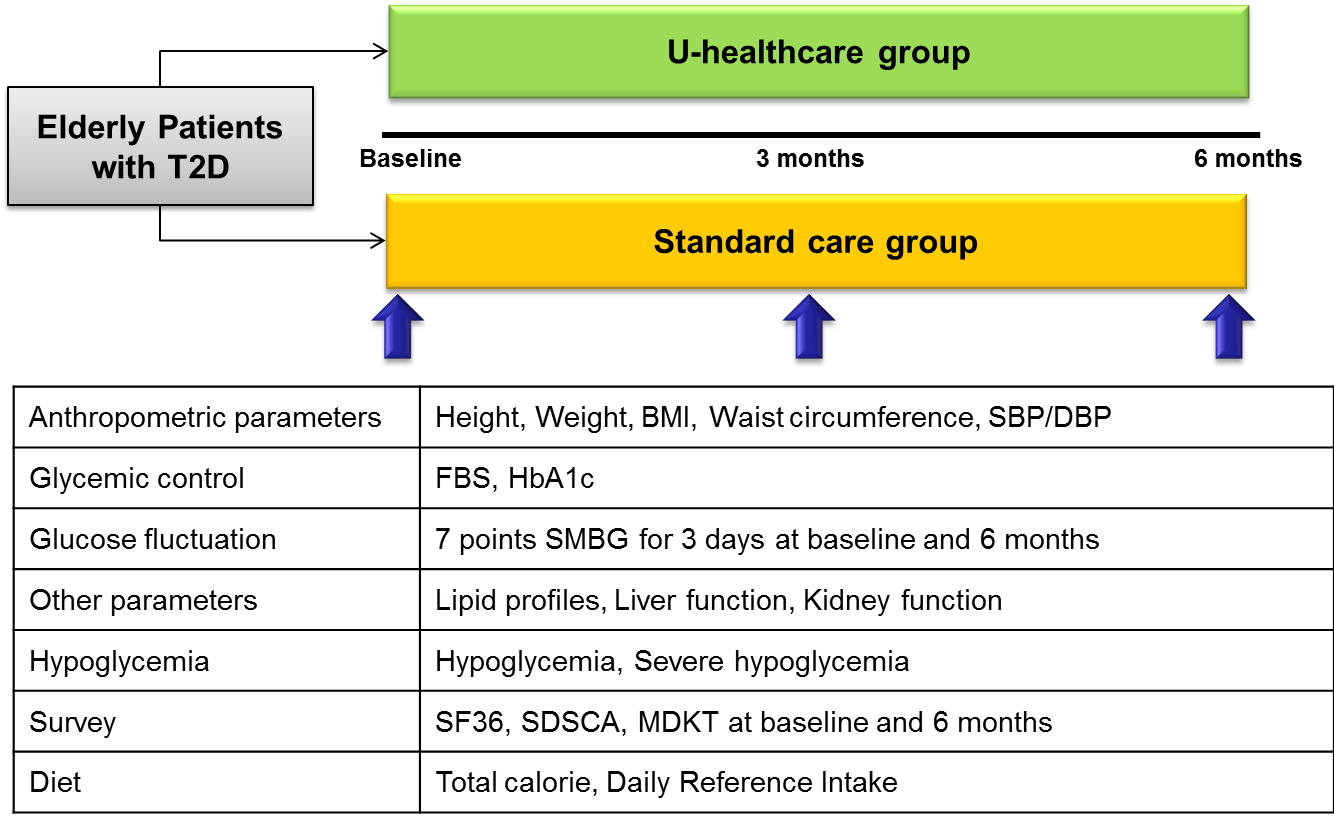
**

**Figure 1. Study scheme and parameters measured at each visit**

**(1) Standard care group**

Patients in the Standard Care group are recommended to measure their blood glucose level at least seven times a week (three or more times fasting, three or more times postprandially, and once or more times at bedtime).

**(2) u-healthcare group**

Participants in the u-healthcare group are educated to use the u-healthcare service using the voice inception technique. They are guided to check their blood glucose levels at the same frequency as the standard care group. All subjects in this study are instructed to complete a 3-day food record (two weekdays and one day on a weekend) at the baseline visit and at the end of the study. Tailored messages automatically generated from the CDSS rule engine are transmitted to their mobile phones immediately.

**2. Procedures**

For both groups, pertinent diabetes education including a therapeutic lifestyle change program are provided to standardize each patient’s understanding of diabetes management according to the recommendations of the American Diabetes Association (ADA) and the Korean Diabetes Association (18,19).

All participants are instructed to measure their blood glucose levels at least seven times a week (three or more times fasting, three or more times postprandially, and once or more at bedtime). Each patient is advised to use their own glucometer.

The patients in the u-healthcare group are educated to report their glucose levels via phone call equipped with voice inception technique. With this device, their glucose levels are transferred to the main server in the u-healthcare center (**Figure 2**). Tailored messages automatically generated from the clinical decision support system (CDSS) rule engine are transmitted to their mobile phone and website immediately.

**(1) Standard care group**

Patients in the Standard care group are instructed to measure their blood glucose levels at least seven times a week (three or more times fasting, three or more times postprandially, and once or more at bedtime). Each patient is advised to use their own glucometer.

The staff members in the u-healthcare center assess each patient’s glucose control status, average caloric intake, and the amount of regular physical activity. Based on these data, an individualized glycemic target goal is given to each participant. The patients in both groups are also equally educated about diet and exercise based on their previous dietary patterns and physical activity levels.

**(2) u-healthcare group**

Participants in the u-healthcare group are educated to use the u-healthcare service using the voice inception technique. They are guided to check their blood glucose levels at the same frequency as the standard care group. The scheme of the u-healthcare service used in this study is as follows (**Figure 2**). Participants are asked to send their health data such as blood glucose level, body weight, exercise, diet, and medication adherence to the u-healthcare center through the auto response system (ARS) or touch pad system (text to speech, TTS) with a mobile phone or a landline. When the registered patients enter their health data using the ARS or touch pad system, it is recognized by the interactive voice response (IVR) system. The patients’ data are analyzed by the CDSS, which is determined by each patient’s clinical institution when he or she is registered at the study. Finally, the patients receive tailored feedback messages by voice or text messages, which are generated automatically through the CDSS rule engine for the data that they entered (**Figure 2**). Alternatively, patients can also log in to the u-healthcare Web site (online u-healthcare center). When they enter their health data in their own page, they can receive tailored messages in the same way as the voice inception method.

For long-term management, an individualized health report is provided as an analysis of each individual’s cumulative data and corresponding messages about the management goal. Detailed information about diabetes management, recommended dietary pattern, and exercise type, and calorie consumption is provided on the online Web pages. Each person can also consult with healthcare professionals through an online bulletin board managed by the u-healthcare center. The patients can call the u-healthcare center when there is an emergency.


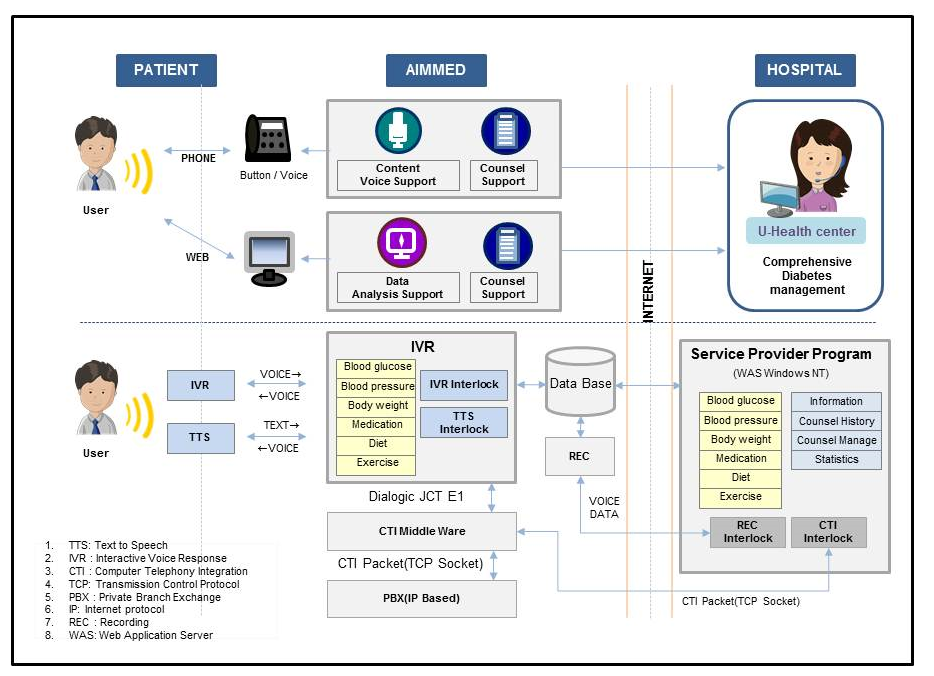


**Figure 2. Scheme of the u-healthcare service using voice-recognition IT solution**

**1) Voice-recognition Interactive Voice Response (IVR)**

Interactive Voice Response (IVR) is an automated telephony system that interacts with callers, gathers information and routes calls to the appropriate recipient. An IVR system allows patients to communicate with clinicians asynchronously using a landline or mobile telephone. Patients are given a toll-free number to use the IVR system in this study. At the beginning of each call, patients’ identities are verified using their self-reported birth date. When they use IVR to call, they hear recorded messages and respond to queries using voice-recognition or their touch-tone keypad. Calls use tree-structured algorithms to present recorded queries and specific information. Based on their responses, patients receive tailored messages for managing blood glucose, physical activity, and diet.

An IVR system consists of telephony equipment, software applications, a [database](http://searchsqlserver.techtarget.com/definition/database) and a supporting [infrastructure](http://searchdatacenter.techtarget.com/definition/infrastructure). In this study, Nuance recognizer 9 (Burlington, MA, USA) of the speaker independent continuous speech recognition engine is applied to our u-healthcare system using speech recognition and text to speech (TTS) output technology, which is a patent of Aimmed corporation Ltd. (Seoul, Korea) **(Figure 3)**. Variety of voice data is collected and tuned to improve the voice-recognition rate for the natural sound output. The system has a user-interface environment of the speech which consists of voice-recognition module through voice-recognition server and voice transmission module through a TTS recognition server. Voice-recognition IVR is used as a method of collecting health data in telemedicine services. It has several advantages; 1) it does not require the purchase of certain monitoring devices which are essential to the existing telemedicine system. Instead, patients can use their own glucometers. 2) Over the phone without the need for internet connection or operation of the equipment, the elderly patients can easily input their measurements and receive analysis results with voice **(Figure 3).**

**
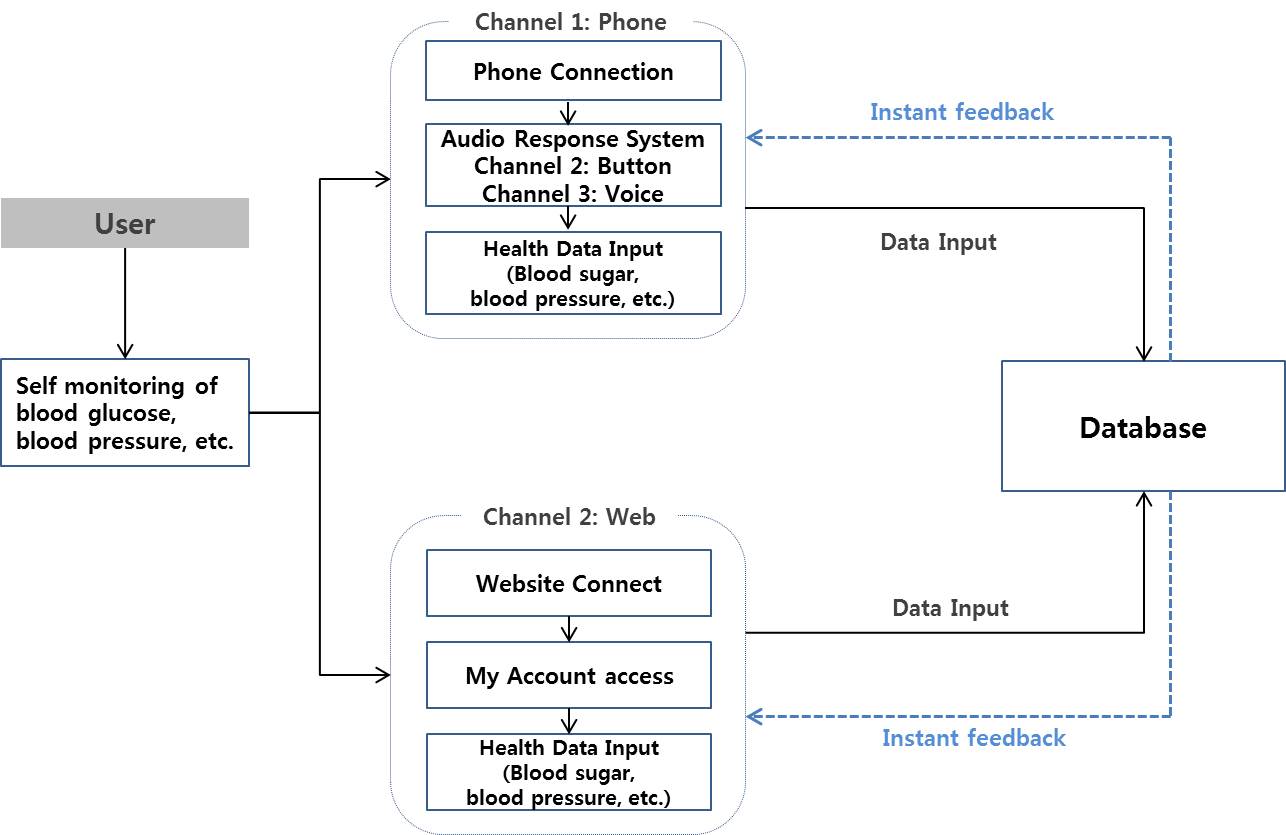
**

**Figure 3. Schematic representation of health management system using voice recognition and text to speech output technology.** Two types of health data input channels; 1) Channel 1 using mobile phone or landline: It is possible to enter health data through voice with ARS or touch pad-tone. 2) Channel 2 using the website: Conventional way where users log-in to the disease management website by typing in their information directly in patient’s administration page.

**2) CDSS based u-healthcare service**

Clinical Decision Support System (CDSS) is a health data analysis logic that was developed by experts in diabetes. It analyzes automatically the health data entered by website, telephone (buttons and voice) in consideration of each management target and input data trends of individuals’ and provides immediate feedback. The CDSS rule engine is based on the clinical practice recommendations of the American Diabetes Association and the Korean Diabetes Association {Korean Diabetes Association, 2007 181 /id;American Diabetes Association, 2010 193 /id}.

The CDSS-generated messages are patient-specific, for example:

1) If a fasting glucose level is 90 mg/dL: → Your blood sugar level is within normal range. Please maintain diet and exercise.

2) If a postprandial glucose level is 250 mg/dL: →Your blood sugar level is high. Postprandial blood glucose level should be within 90 - 200 mg/dL. Watch your diet and/or do more exercise. Make sure you take drugs regularly.

3) If the patient’s fasting glucose level is 65 mg/dL: → Your blood sugar level is low (hypoglycemia level). Please have snacks containing sugar such as orange juice or candy, and then measure your blood glucose level after 15 minutes. If it is still less than 70 mg/dL, repeat this process again.

- Weekly and monthly based messages are also given to the participants.
- If fasting glucose levels are >130 mg/dL more than twice a week, a message to change the patient’s lifestyle or take medication properly.
- If fasting glucose levels are <70 mg/dL more than twice a week, a message encouraging to check calorie intake, exercise habits, medication adherence and consider contact the medical staff.
- Specific messages are given to the participants whose blood glucose levels are in hypoglycemic ranges.
- If glucose level falls <50 mg/dL, a notification is sent to clinicians and patients to ensure appropriate action to treat hypoglycemia.

Additionally, an automatic reporting function analyzes the trends of cumulative measurements and medication adherence in a set period that the user input considering individual targets. The system is also designed to remind patients to record or input their data if information is not received over a week.

If a patient is in a condition where medical staff assistance is required, an SMS is sent to medical staff immediately. If further responses from the patient are not entered within one hour, a u-healthcare staff gives him or her an outbound call.

**3) The u-healthcare center**

The u-healthcare center staff members consist of doctors, nurses, nutritionists, and engineers. The doctors have experiences in diabetes management for more than 5 years. The nurses and nutritionists are certified for diabetes education.

**A. Operating system**

The Computer Telephony Integration (CTI) program that the u-healthcare center staff members use for operating u-healthcare system consists of the following; 1) User management: User information management, health data management, etc., 2) Statistics management: Service operation statistics, health data statistics analysis, etc., and 3) Operations management: Medical agent management, etc. (**Figure 4**).


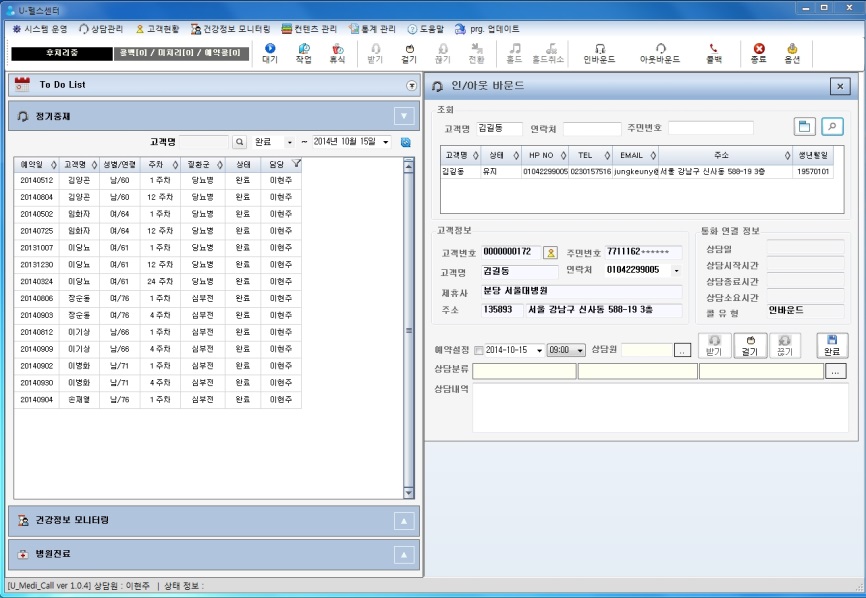


**Figure 4. The Computer Telephony Integration (CTI) for operating u-healthcare system**

**B. u-healthcare website: online u-healthcare Center**

For this study, we created a dedicated website containing a glucose control section, diet control section, physical activity section, and an integrated management section (**Figure 5**). Participants in the u-healthcare group are educated how to log on and use the website.

**(1) Intro page**

Upon logging in the website, patients can see most recent data of their health information; fasting and postprandial glucose concentrations, HbA1c, body weight, diet intake, and physical activity. They can also check their medication adherence, online consultation history, and other medical history that they input previously.


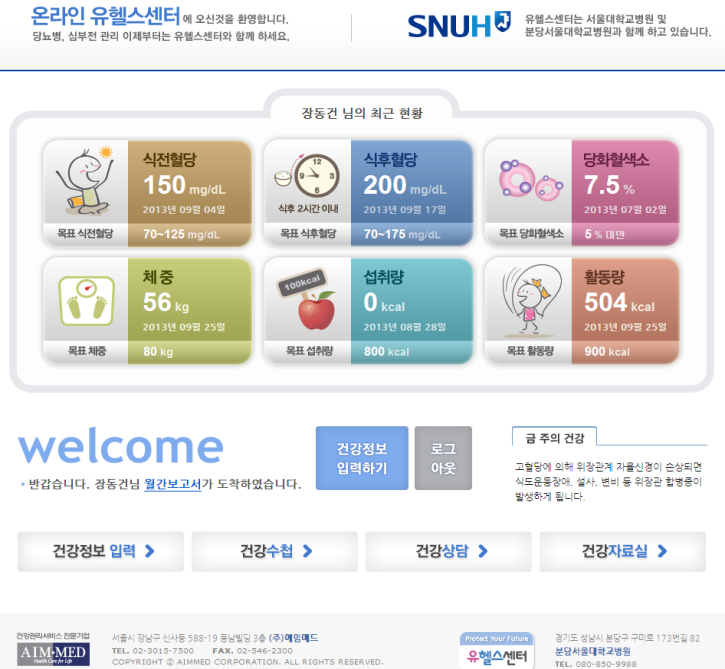

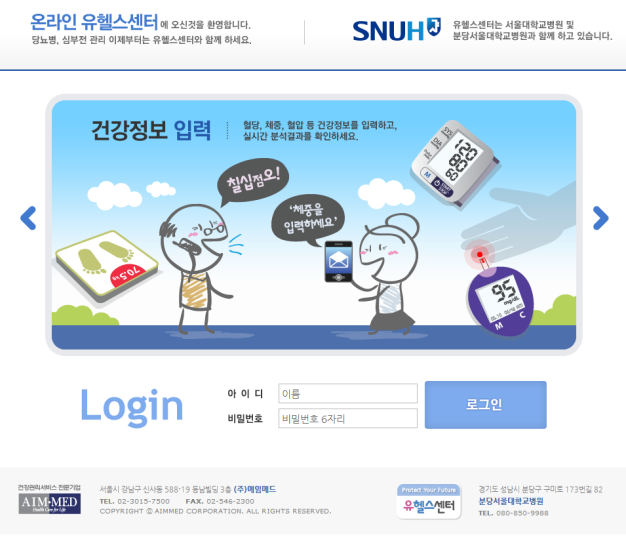


**Figure 5. The u-healthcare website: (A) Log in page (B) First page showing the latest health data after longing in.**

**(2) Health data page**

Participants are guided to enter their health information such as fasting and postprandial glucose levels, body weight, blood pressure, pulse rate, medications, calorie intake and physical activity **(Figure 6A).** They can also check real-time feedback regarding the data that they input **(Figure 6B)**.


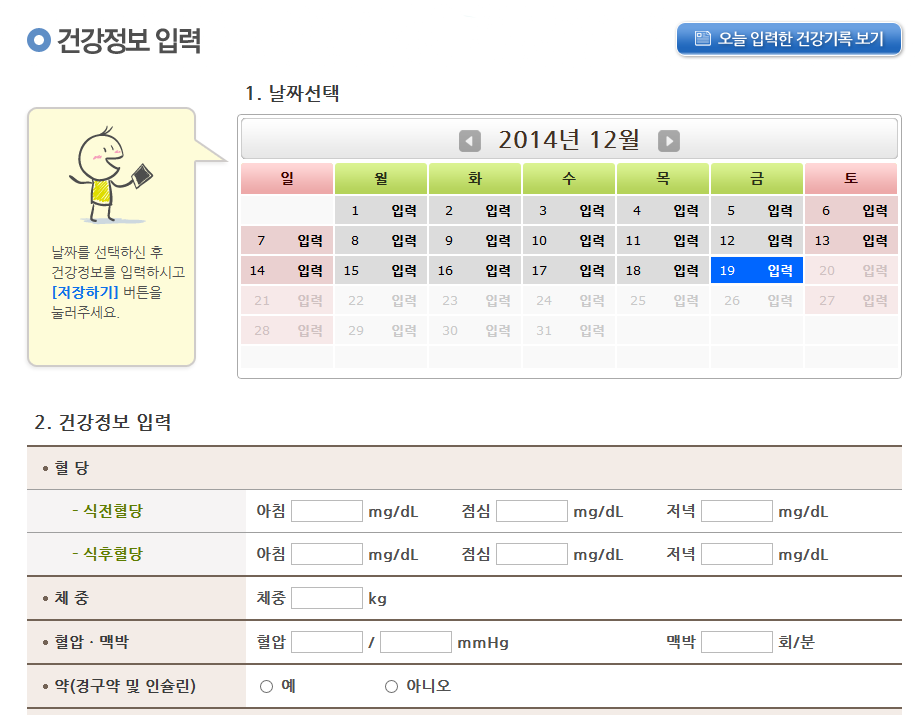

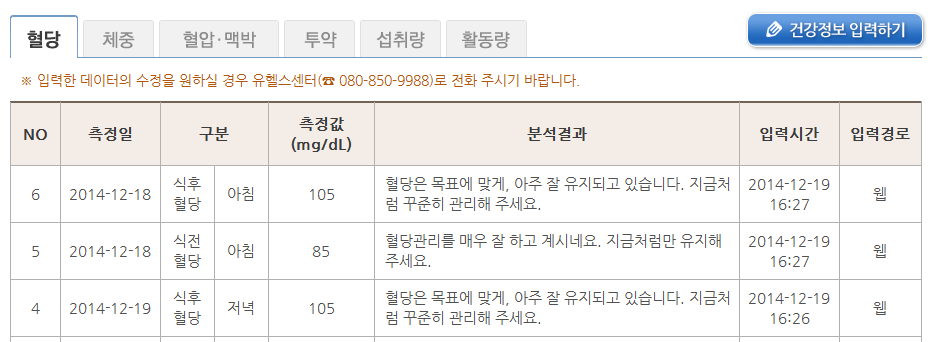


**Figure 6. Health data page: (A) Input page (B) Real-time feedback**

Comprehensive analysis and recommendations are provided for weekly and monthly data of blood glucose, calorie intake and physical activity, and medication adherence based on CDSS (**Figure 7**). In the weekly reports, the average values of blood glucose before and after meals for one week are transmitted automatically with a short message to patients. In the monthly reports, the average blood glucose level for one month with previous monthly-based averages, the frequency of reaching the target range, and hypoglycemia are transmitted automatically to the patients.


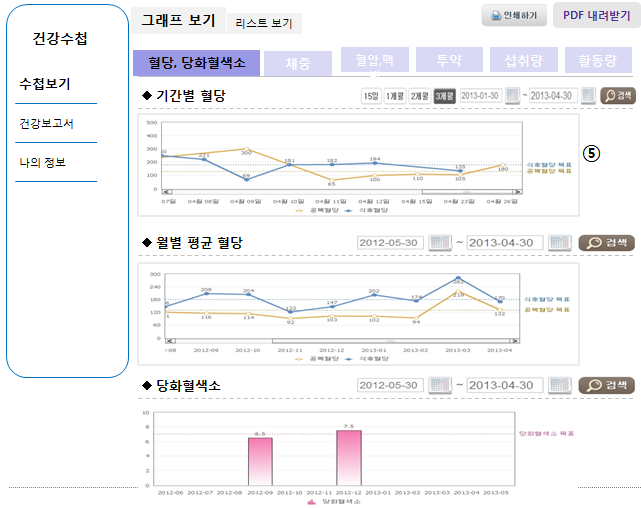

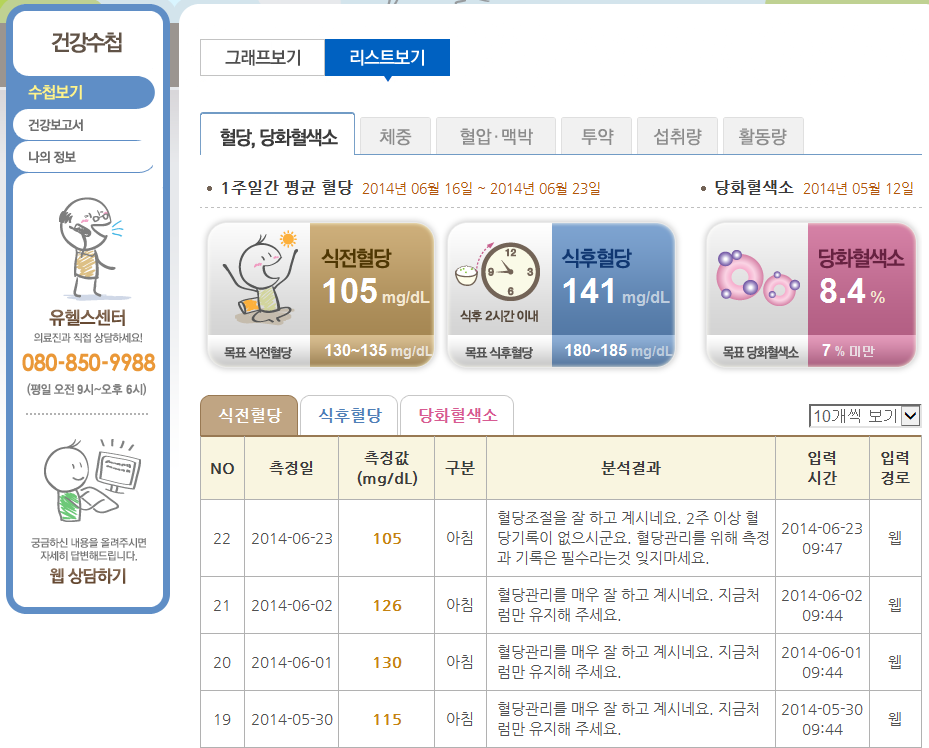


**Figure 7. Trend of fasting and postprandial glucose values and HbA1c levels (A) and average values of and feedback messages for fasting and postprandial glucose and HbA1c levels**

**(3) Physical activity page**

In consideration of each patient’s disease and weight, the recommended daily activity calorie regimen is guided individually. The daily calorie activity recommendation applies only to patients who received the weight loss push.

Recommended calorie calculation is as follows:

- Daily recommended activity calories = weight to lose (kg) x7,000kcal / 28 days / 2
- Calories burned = 1.05 x METs(metabolic equivalents) x weight x Exercise time (hour)

For patients who do not need to lose weight, exercise three to five times per week is recommended. If data on physical activity and time spent is input, calories burned from the physical activity are calculated (**Figure 8**). Through the CDSS, a report on the activity input for 1 week is generated and sent to the patient**.**


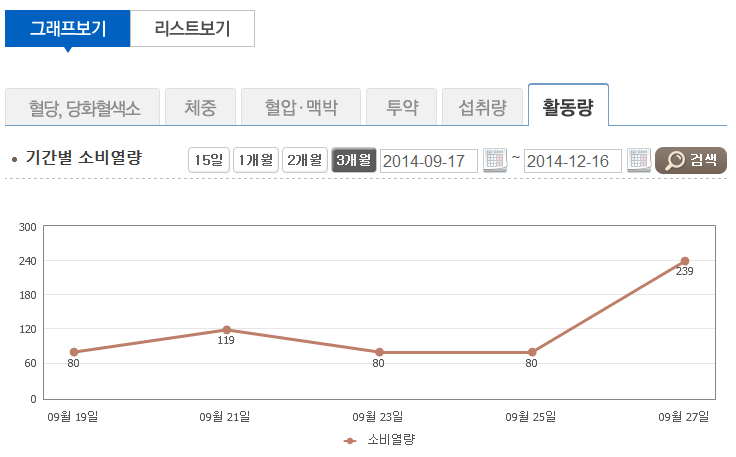

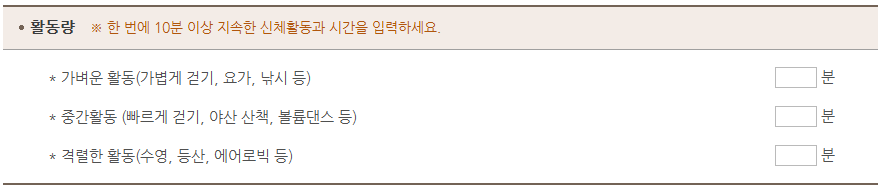


**Figure 8.** (A) Physical activity input page. The activity levels are divided into light activity, medium activity, intense activity and METs per activity was based on the International Physical Activity Questionnaires (IPAQ). (B) Physical activity output page.

**(4) Feedback for caloric intake**

For dietary feedback, participants are educated to give information on their food intake via phone. They are also able to upload a food diary or a picture of a food on the health data input page on the Web site. In the Web site, a list of each participant’s favorite food is uploaded by a dietician to help patient click on what and how much they eat. Nutritionists directly analyze the customer-specific dietary problems through the analysis program called CAN-Pro 4.0 (Korean nutrition Society). The results from the CAN-Pro software are displayed automatically on the Web site, and the participants can find detailed information about their dietary pattern or, if necessary, a nutritionist directly provides direct consultations via the phone. Nutritionists prescribe the recommended daily calorie intake taking into account the patient’s weight and disease and monitor eating habits in the following ways: Face-to-face dietary surveys are performed at baseline and end of study. During the study period, a user is educated to give information via phone about food intake. Nutritionists in the u-healthcare center check the recorded file and give them dietary advice, if necessary. Nutritionists advise the subjects who are overweight or obese to consume less carbohydrate and saturated fat intakes and for those who are underweight to increase protein intake. The participants who have high sodium intakes are educated to lessen the sodium intakes to the optimal level. Furthermore, participants are encouraged to modify undesirable eating behaviors~~,~~ such as irregular dietary intakes, late-night snacking, and binge eating according their nutritional reports. On the 12th week~~s~~ and 24th week~~s~~, the nutritional evaluation reports are sent to the participants (**Figure 9**).


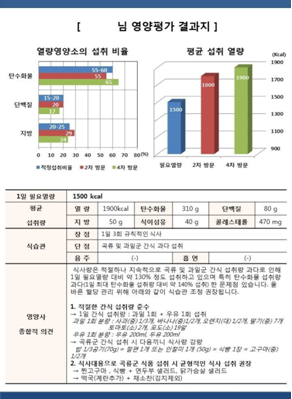


**Figure 9. A sample of nutritional evaluation report**

**(5) A webpage for information about diabetes and its management (Health Library)**

We created a Health Library on the website to provide the necessary guide to diabetes management and healthy lifestyles (**Figure 10**). In this website, participants can find detailed information about diabetes mellitus and several guidelines for diet and exercise.


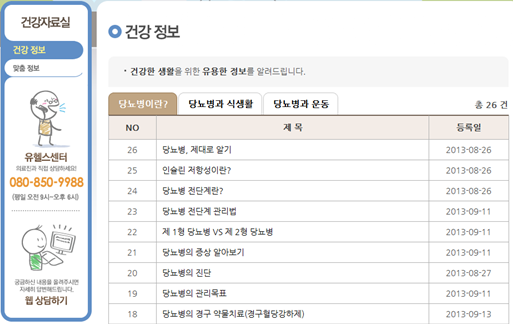


**Figure 10. The Health library webpage for information about diabetes and its management**

**(6) Health counseling services**

Patients are given a toll-free number where they can contact u-healthcare staff members for counseling about disease management and any questions on u-healthcare system (**Figure 11**). If a patient is in a condition needed to be connected to the medical staff, a u-healthcare staff gives him or her an outbound call. If a patient is in a condition where medical staff assistance is required, a u-healthcare staff gives him or her an outbound call.

**
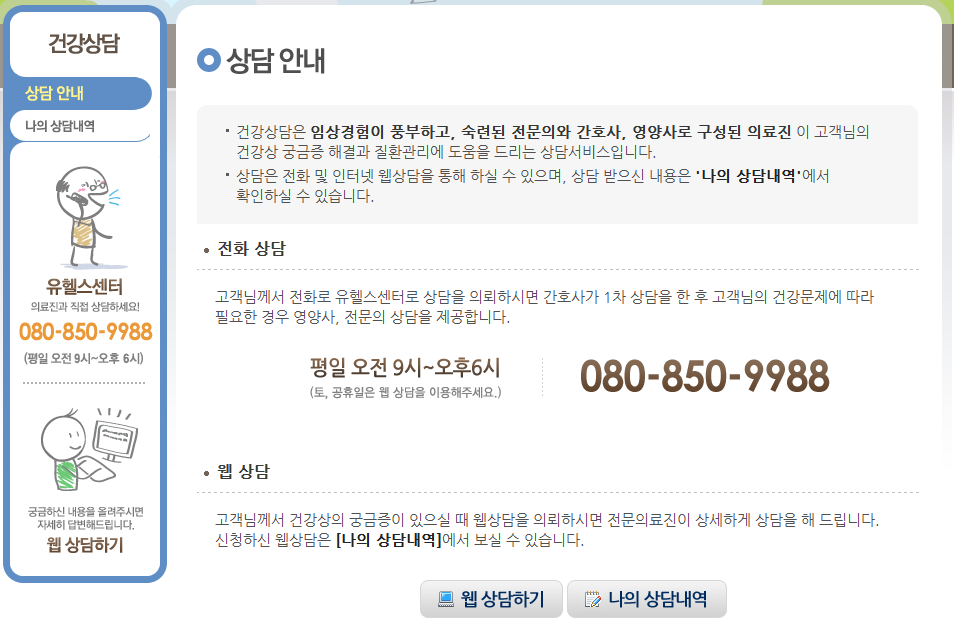
**

**Figure 11. The website for information of counseling service via phones and website.**

References

1. Ding H, Moodley Y, Kanagasingam Y, Karunanithi M. A mobile-health system to manage chronic obstructive pulmonary disease patients at home. Conf Proc IEEE Eng Med Biol Soc 2012;2012:2178-2181

2. Steventon A, Bardsley M, Billings J, et al. Effect of telehealth on use of secondary care and mortality: findings from the Whole System Demonstrator cluster randomised trial. BMJ 2012;344:e3874

3. de JT, Gurol-Urganci I, Vodopivec-Jamsek V, Car J, Atun R. Mobile phone messaging for facilitating self-management of long-term illnesses. Cochrane Database Syst Rev 2012;12:CD007459

4. Alasaarela E, Oliver NS. Wireless solutions for managing diabetes: A review and future prospects. Technol Health Care 2009;17:353-367

5. Perez-Ferre N, Galindo M, Fernandez MD, et al. A Telemedicine system based on Internet and short message service as a new approach in the follow-up of patients with gestational diabetes. Diabetes Res Clin Pract 2010;87:e15-e17

6. Mulvaney SA, Rothman RL, Wallston KA, Lybarger C, Dietrich MS. An internet-based program to improve self-management in adolescents with type 1 diabetes. Diabetes Care 2010;33:602-604

7. Cho JH, Chang SA, Kwon HS, et al. Long-term effect of the Internet-based glucose monitoring system on HbA1c reduction and glucose stability: a 30-month follow-up study for diabetes management with a ubiquitous medical care system. Diabetes Care 2006;29:2625-2631

8. Lim S, Kang SM, Shin H, et al. Improved glycemic control without hypoglycemia in elderly diabetic patients using the ubiquitous healthcare service, a new medical information system. Diabetes Care 2011;34:308-313

9. Lim S, Kang SM, Shin H, et al. Improved glycemic control without hypoglycemia in elderly diabetic patients using the ubiquitous healthcare service, a new medical information system. Diabetes Care 2011;34:308-313

10. Kang SM, Kim MJ, Ahn HY, et al. Ubiquitous healthcare service has the persistent benefit on glycemic control and body weight in older adults with diabetes. Diabetes Care 2012;35:e19

11. Hu FB, Manson JE, Stampfer MJ, et al. Diet, lifestyle, and the risk of type 2 diabetes mellitus in women. N Engl J Med 2001;345:790-797

12. Knowler WC, Barrett-Connor E, Fowler SE, et al. Reduction in the incidence of type 2 diabetes with lifestyle intervention or metformin. N Engl J Med 2002;346:393-403

13. Li G, Zhang P, Wang J, et al. The long-term effect of lifestyle interventions to prevent diabetes in the China Da Qing Diabetes Prevention Study: a 20-year follow-up study. Lancet 2008;371:1783-1789

14. Cardiovascular Effects of Intensive Lifestyle Intervention in Type 2 Diabetes. N Engl J Med 2013;369:145-154

15. Gerstein HC, Miller ME, Byington RP, et al. Effects of intensive glucose lowering in type 2 diabetes. N Engl J Med 2008;358:2545-2559

16. Patel A, MacMahon S, Chalmers J, et al. Intensive blood glucose control and vascular outcomes in patients with type 2 diabetes. N Engl J Med 2008;358:2560-2572

17. Munshi MN, Maguchi M, Segal AR. Treatment of type 2 diabetes in the elderly. Curr Diab Rep 2012;12:239-245

18. Korean Diabetes Association: Treatment Guideline for Diabetes. Korean Diabetes Association 2007;

19. Standards of medical care in diabetes-2010. Diabetes Care 2010;33 Suppl 1:S11-S61

20. Cui HS, Ahn IS, Byun YS, et al. Dietary pattern and nutrient intake of korean children with atopic dermatitis. Ann Dermatol 2014;26:570-575

21. Huang IC, Hwang CC, Wu MY, Lin W, Leite W, Wu AW. Diabetes-specific or generic measures for health-related quality of life? Evidence from psychometric validation of the D-39 and SF-36. Value Health 2008;11:450-461

22. Han CW, Lee EJ, Iwaya T, Kataoka H, Kohzuki M. Development of the Korean version of Short-Form 36-Item Health Survey: health related QOL of healthy elderly people and elderly patients in Korea. Tohoku J Exp Med 2004;203:189-194

23. Toobert DJ, Hampson SE, Glasgow RE. The summary of diabetes self-care activities measure: results from 7 studies and a revised scale. Diabetes Care 2000;23:943-950

24. Fitzgerald JT, Funnell MM, Hess GE, et al. The reliability and validity of a brief diabetes knowledge test. Diabetes Care 1998;21:706-710

25. Service FJ, Molnar GD, Rosevear JW, Ackerman E, Gatewood LC, Taylor WF. Mean amplitude of glycemic excursions, a measure of diabetic instability. Diabetes 1970;19:644-655

26. Lim S, Kang SM, Shin H, et al. Improved glycemic control without hypoglycemia in elderly diabetic patients using the ubiquitous healthcare service, a new medical information system. Diabetes Care 2011;34:308-313

27. Standards of medical care in diabetes--2014. Diabetes Care 2014;37 Suppl 1:S14-S80

28. Snell-Bergeon JK, Wadwa RP. Hypoglycemia, diabetes, and cardiovascular disease. Diabetes Technol Ther 2012;14 Suppl 1:S51-S58

29. McCoy RG, Van Houten HK, Ziegenfuss JY, Shah ND, Wermers RA, Smith SA. Increased mortality of patients with diabetes reporting severe hypoglycemia. Diabetes Care 2012;35:1897-1901

30. Bremer JP, Jauch-Chara K, Hallschmid M, Schmid S, Schultes B. Hypoglycemia unawareness in older compared with middle-aged patients with type 2 diabetes. Diabetes Care 2009;32:1513-1517

31. Lee HJ, Lee SH, Ha KS, et al. Ubiquitous healthcare service using Zigbee and mobile phone for elderly patients. Int J Med Inform 2009;78:193-198

32. Cho JH, Chang SA, Kwon HS, et al. Long-term effect of the Internet-based glucose monitoring system on HbA1c reduction and glucose stability: a 30-month follow-up study for diabetes management with a ubiquitous medical care system. Diabetes Care 2006;29:2625-2631

33. Yoo HJ, Park MS, Kim TN, et al. A Ubiquitous Chronic Disease Care system using cellular phones and the internet. Diabet Med 2009;26:628-635

34. Lim S, Kang SM, Shin H, et al. Improved glycemic control without hypoglycemia in elderly diabetic patients using the ubiquitous healthcare service, a new medical information system. Diabetes Care 2011;34:308-313

35. Chaudhry SI, Mattera JA, Curtis JP, et al. Telemonitoring in patients with heart failure. N Engl J Med 2010;363:2301-2309

36. World Medical Association declaration of Helsinki. Recommendations guiding physicians in biomedical research involving human subjects. JAMA 1997;277:925-926
